# **Effect of PLC-β1/CaM signaling pathway mediated by AT1R on the occurrence and development of hepatocellular carcinoma**

**DOI:** 10.1186/s12935-021-02261-8

**Published:** 2021-11-02

**Authors:** Zhou-wei Xu, Na-na Liu, Xing-yu Wang, Bai-cheng Ding, Hai-feng Zhang, Ying Li, Wu-yi Sun, Wei Wei

**Affiliations:** 1 Department of Emergency Surgery, The First Affiliated Hospital of Anhui Medical University, Jixi Road, Hefei, 230022 Anhui People’s Republic of China; 2 Key Laboratory of Anti-Inflammatory and Immune Medicine, Ministry of Education, Anhui Collaborative Innovation Center of Anti-Inflammatory and Immune Medicine, Institute of Clinical Pharmacology, Anhui Medical University, Meishan Road, Hefei, 230032 Anhui People’s Republic of China; 3 Department of Clinical Medical, the First Clinical Medical College of Anhui Medical University, Meishan Road, Hefei, 230032 Anhui People’s Republic of China

**Keywords:** Hepatocellular carcinoma, AT1R, PLC-β1, CaM, Migration, Invasion

## Abstract

**Objective:**

To study the roles of AT1R, PLC-β1, CaM and other related signal molecules in the formation and development of hepatocellular carcinoma (HCC) and their correlation.

**Methods:**

ELISA and immunohistochemistry were used to analyze the expressions of target proteins in serum and liver tissue of HCC patients, and the correlation between AT1R, PLC-β1 and CaM and postoperative survival status of patients was followed up and determined. CCK-8 method was used to screen the doses of Ang II and candesartan sensitive to HepG2 and HCCLM3 cells. Transwell experiment was used to observe the effects of different drugs on the migration and invasion activity of HCC cells. Meanwhile, flow cytometry and Western blot were used to detect the expression levels of AT1R, PLC-β1 and CaM in the cells. Then PLC-β1 siRNA was selected to transfect HCC cells, so as to further clarify the mechanism of the above signal proteins. HepG2 cells were inoculated under the hepatic capsule of mice to induce the formation of HCC in situ. Ang II and candesartan were used to stimulate HCC mice to observe the difference in liver appearance and measure the liver index. Finally, ELISA and immunofluorescence experiments were selected to analyze the levels of target proteins in mouse serum and liver tissue.

**Results:**

The expression levels of target proteins in serum and liver tissue of HCC patients were significantly increased, and the postoperative survival time of patients with high expression of AT1R, PLC-β1 or CaM was obviously shortened. Ang II and candesartan could significantly promote and inhibit the motility of HCC cells, and had different effects on the levels of AT1R, PLC-β1 and CaM in cells. However, in hepatocellular carcinoma cells transfected with PLC-β1 siRNA, the intervention ability of drugs was obviously weakened. Ang II could significantly promote the formation and progression of mouse HCC, while candesartan had the opposite effect. Meanwhile, medications could affect the expressions of target proteins in mouse serum and liver tissue.

**Conclusion:**

AT1R, PLC-β1 and CaM may be risk factors affecting the formation and prognosis of HCC, and the PLC-β1/CaM signaling pathway mediated by AT1R is an important way to regulate the migration and invasion activity of HCC cells.

**Supplementary Information:**

The online version contains supplementary material available at 10.1186/s12935-021-02261-8.

## Introduction

Primary hepatocellular carcinoma (HCC) is one of the common malignant tumors in the digestive system. Currently, the incidence rate of HCC presents an upward trend around the world. In China, the prevalence rate of HCC exceeds 20/1,00,000, while the 5-year survival rate is only about 10%, making it the third largest malignant tumor in mortality, behind lung cancer and gastric cancer [1–3]. The etiology and pathogenesis of HCC are still undetermined. Due to its hidden onset and lack of early symptoms, most patients are already in the advanced stage of the disease when they are diagnosed, and even have advanced manifestations such as acute liver failure, HCC rupture and hemorrhage, and distant tumor metastasis [4]. Even more seriously, patients with poorly differentiated HCC have a significantly increased capacity for tumor cell invasion and angiogenesis in vivo, and a high recurrence rate after surgical treatment, leading to an obvious increase in mortality in these patients [5]. The formation and development of HCC are closely related to abnormal cell signal transduction pathways, and regulating the expression of signal proteins can affect the proliferation and invasion of HCC cells. Therefore, the exploration on related molecules such as AT1R, PLC-β1 and CaM can provide theoretical basis for finding novel drug targets.

**Fig. 1:**
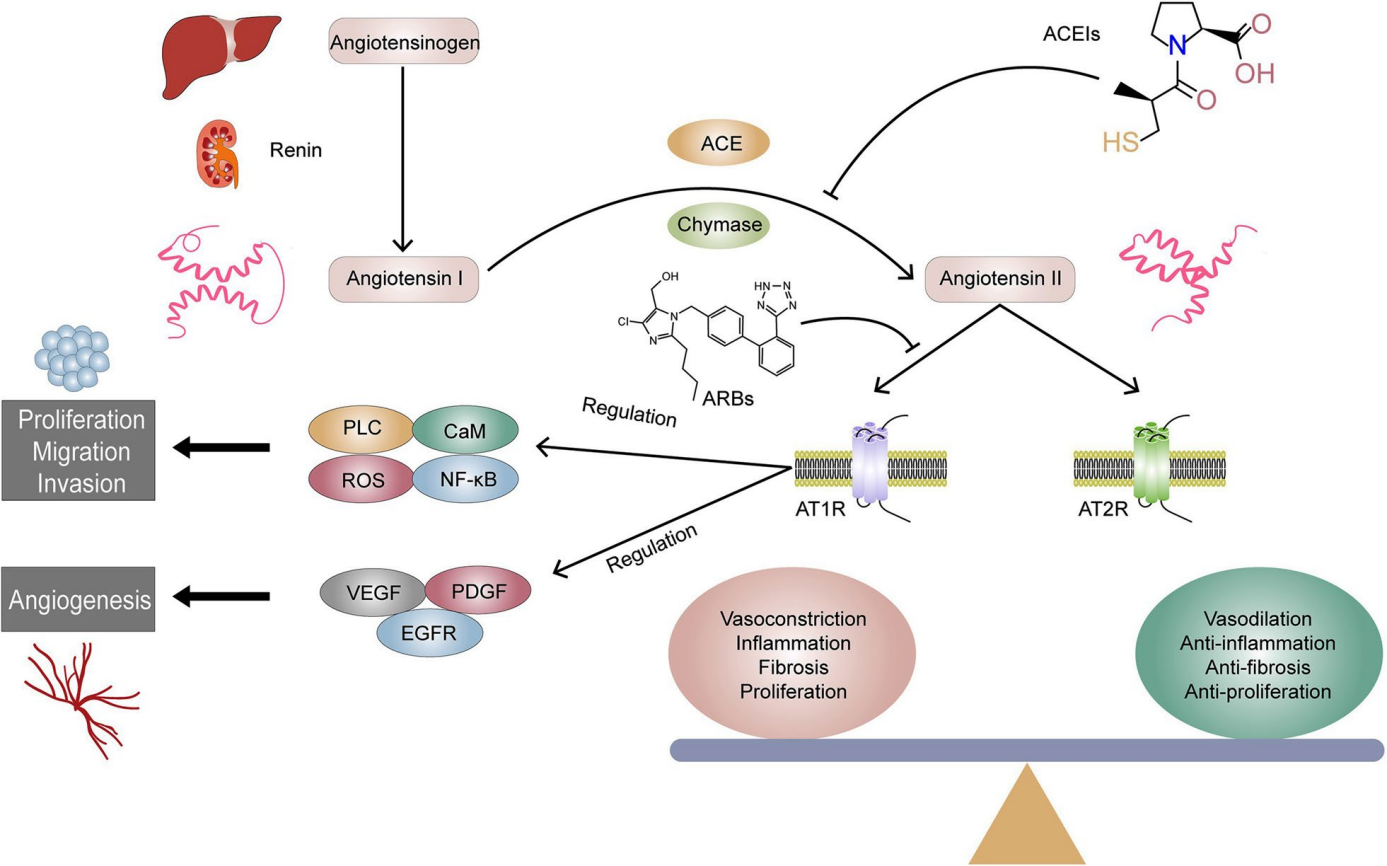
Schematic diagram of Ang II exerting different biological functions through corresponding receptors

Angiotensin II (Ang II) is an important vasoactive substance in human body, which can produce biological effects such as vasoconstriction, pro-inflammation, fibrosis and cell proliferation mediated by angiotensin II type 1 receptor (AT1R), while angiotensin II type 2 receptor (AT2R) may have completely opposite biological effects (Fig. 1) [6–8]. Ang II can be produced a large number in tumor tissues and up-regulate the expression of AT1R, further regulate the activities of signal proteins such as phospholipase-C (PLC), reactive oxygen species, nuclear factor-κappa B and nitric oxide, and affect the growth and metastasis of tumor cells [9, 10]. AT1R is the main angiotensin receptor in liver tissue, and its abnormal expression has been proved to be closely related to liver fibrosis and carcinogenesis. Previous experiments by our research group found that the expression levels of Ang II and AT1R in human HCC tissue were significantly increased, which can obviously enhance the proliferation, migration and invasion capabilities of HCC cells [11, 12].

**Fig. 2:**
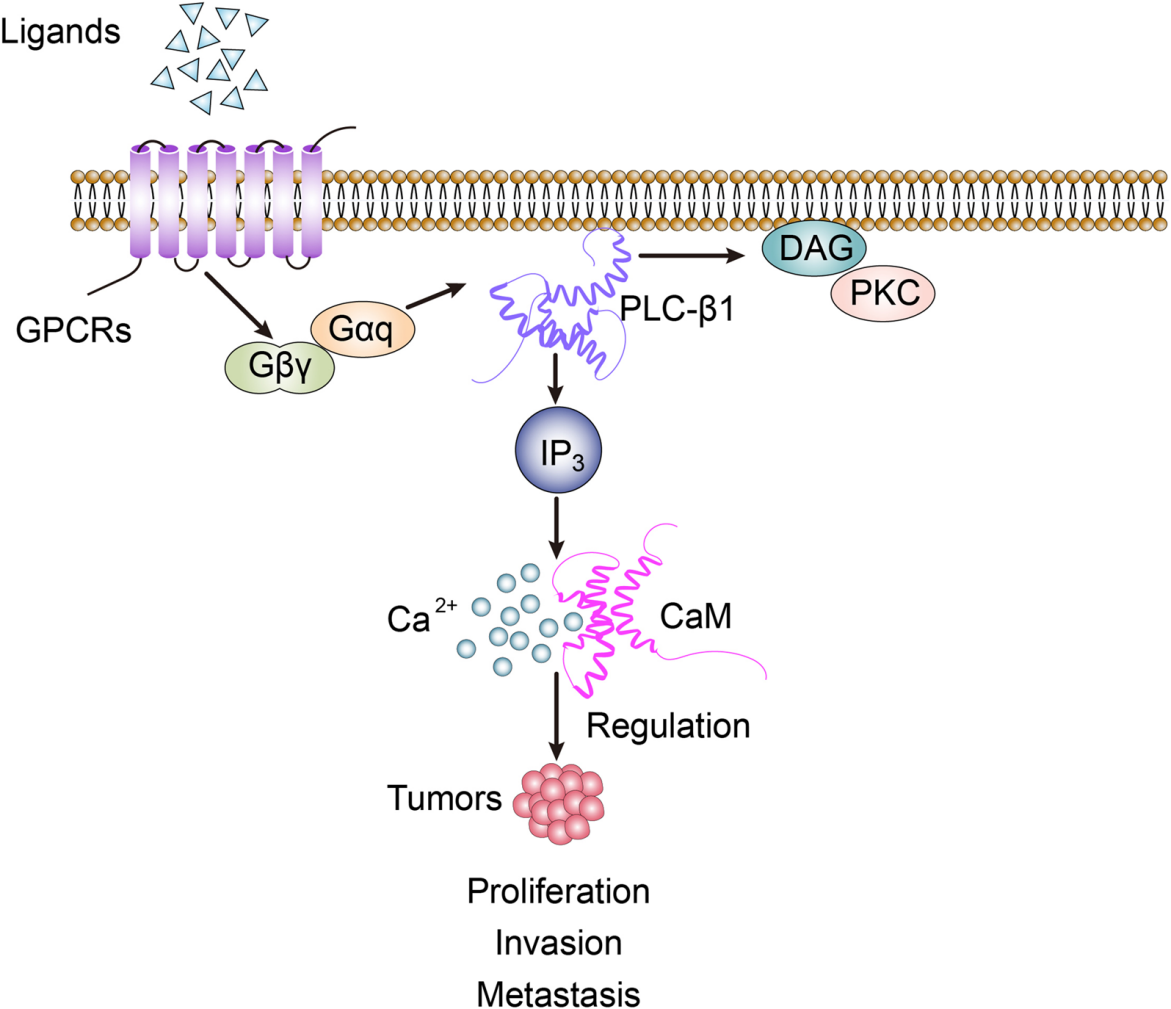
PLC-β1/CaM-related signal transduction involves in the regulation of biological activity of tumor cells

PLC can be divided into 6 categories such as β, γ, δ, ε, ζ and η according to different amino acid sequences. Coupling with GPCRs such as AT1R is the main way of PLC activation. PLC-β subfamily includes PLC-β1–PLC-β4, among which PLC-β1 gene is located on the short arm of chromosome 20, and distributed in human liver, brain, pancreas and vascular smooth muscle tissues [13–16]. Extracellular factors such as Ang II, epinephrine, histamine and bradykinin can bind to corresponding receptors to activate PLC-β1 protein, further promote CaM expression and conformational changes in cells, thus regulating intracellular Ca^2+^ concentration and calcium kinase activity, producing a series of biological effects (Fig. 2) [17]. Recent studies have found that the contents of PLC-β1 and CaM in HCC, colon cancer and breast cancer tissues are significantly increased, which can promote the growth, invasion and movement of tumor cells [18–20].

AT1R, PLC-β1 and CaM are important bioactive molecules involved in the proliferation and invasion of HCC cells. However, the relationship between them remains unclear. This study combines clinical, animal and cell experiments to analyze whether AT1R affects the occurrence and development of HCC through mediating PLC-β1/CaM signaling pathway, and lays a theoretical foundation for further exploring the roles of AT1R and its related signals in the formation and prognosis of HCC in the future.

## Materials and methods

### Clinical specimens

In the study, the clinical data, serum and liver tissue specimens of 65 HCC patients who underwent surgical treatment in the Department of Hepatobiliary Surgery of the First Affiliated Hospital of Anhui Medical University from 2014 to 2015 were collected, and the serum of 82 patients having normal outpatient physical examination in the same period was collected as control. All HCC cases were definitely diagnosed by postoperative pathological examination, and no radiotherapy or chemotherapy was performed before operation. The corresponding research content has obtained the informed consent of patients and approved by the Biomedical Ethics Committee of Anhui Medical University, and conforms to the ethical standards stipulated in Helsinki Declaration for Human Medical Experiments [21].

### Postoperative follow-up

According to the expressions of AT1R, PLC-β1 and CaM in liver tissue of HCC patients, the follow-up end point is the time of death or to 5 years after operation. It is suggested that patients should carry out reexamination of liver function, hepatitis B virus DNA quantification, alpha fetoprotein (AFP) and liver enhanced CT every 3–6 months in our hospital, focusing on judging whether patients have symptoms such as tumor recurrence or metastasis. Kaplan–Meier survival curve was drawn by analyzing the postoperative follow-up results of patients, and the relationship between the corresponding target protein and survival status of patients was observed, so as to evaluate the risk factors affecting the prognosis of HCC.

### Mouse HCC model

BALB/c female mice born 5 weeks ago were selected to establish HCC in situ model. All mice came from Animal Experimental Center of Anhui Medical University and were randomly divided into model group and control group according to their body weight. 0.05 mL of human HepG2 cells (Shanghai Cell Bank) with a density of 1 × 10^8^ cells/mL were inoculated under the liver capsule of mice to induce HCC formation, while the control group was injected with the same volume of normal saline (NS). In addition, mice with HCC were stimulated with continuous subcutaneous infusion of Ang II (#A9525; 1 μg/kg/min; sigma; St. Louis, Mo, USA) and intragastric administration of candesartan (#889652; 5000 μg/kg/d; Takeda; Osaka, Japan), while NS administration was established as a control. In vivo imaging was performed 1 week, 2 weeks and 3 weeks after administration, and the differences in liver appearance and liver index of mice in each group were observed. All the mice were sacrificed by cervical dislocation after anesthesia.

### Cell culture and transfection

HCC cells HCCLM3 and HepG2 with different metastatic potential were cultured to observe their biological activities. The cell lines were all derived from the Shanghai Cell Bank of the Chinese Academy of Sciences and grew in DMEM medium containing 10% fetal bovine serum (#SH30396; HyClone; Logan, UT, USA). PLC-β1 siRNA and scrambled siRNA (SCBT; Santa Cruz, CA, USA) were used to interfere with HCC cells. The sequence of PLC-β1 siRNA is as follows: 5′-CGAUGACUGUAAGGCGUCUAU-3′, and the sequence of scrambled siRNA is as follows: 5′-AUCGAUAACCGUAACGUUGA-3′. The diluted Lipofectamine 3000 (#L3000008; Invitrogen; Carlsbad, CA, USA) and siRNA reagent were fully mixed in a suitable proportion to form a complex, which was added into the cells to be transfected, and the induction process was completed by continuous incubation for 24–48 h.

### ELISA assay

Human and mouse peripheral blood samples collected by coagulation-promoting tube were centrifuged at 1500 rpm for 15 min, and the collected supernatant was the serum needed for the experiment. Sample wells and control wells were set in the ELISA plate, in which the serum to be tested was added to the sample wells, then horseradish peroxidase (HRP) labeled Ang II and AT1R-Ab primary antibodies (#12000, #52700; CellTrend; Luckenwalde, Germany) were added and incubated in an incubator at 37 ℃ for 1 h. 100 μL substrate chromogenic solution (#DY999; R&D system; Minneapolis, MN, USA) was added to each well to continue incubation in the dark for 15 min. Finally, the OD value of serum in each group was measured by spectrophotometer at 450 nm wavelength.

### HE staining

Human and mouse liver specimens fixed in formalin were embedded in paraffin and sectioned at a thickness of 4 μm, and then soaked in xylene and gradient alcohol to complete tissue dewaxing. Then hematoxylin staining solution and 0.5% eosin alcohol (#C0109; Beyotime; Shanghai, China) were used to stain the nucleus and cytoplasm, and finally the tissue cell morphology was observed under the Olympus BX53 optical microscope (Olympus; Tokyo, Japan). According to Edmondson-Steiner pathological grading method, the differentiation degree, nuclear/cytoplasm ratio and atypia of HCC cells in each group were evaluated, and they were divided into grade I, II, III and IV [22, 23].

### Immunohistochemistry

Paraffin sections of human liver tissue were baked, dewaxed and antigen repaired, and then incubated with 3% hydrogen peroxide (H_2_O_2_) in the dark. Diluted anti-AT1R, anti-PLC-β1 and anti-CaM monoclonal antibodies (#ab124505, #ab182359, #ab45689; Abcam; Cambridge, UK) were added dropwise and placed in a wet box at 4 ℃ overnight. The sections were washed with phosphate buffer solution (PBS), then goat serum and HRP-labeled secondary antibody (#WBKLS0500; Millipore; Bedford, MA, USA) were added in turn, and the tissues were stained with DAB chromogenic solution and hematoxylin staining solution. The difference of yellow or brown granules expression in HCC and para-carcinoma tissues was observed under a 200-fold optical microscope, and the average optical density of the target protein was quantitatively calculated by Image-Pro Plus software.

### Immunofluorescence

Paraffin sections of mouse liver tissue were baked and dewaxed in an incubator at 60 ℃, antigen repair was completed with citrate buffer, and then blocked with goat serum. Diluted anti-AT1R, anti-PLC-β1 and anti-CaM polyclonal primary antibodies (#DF4910, #DF6726, #AF6353; Affinity; Cincinnati, OH, USA) and Cy3 labeled secondary antibody (#ab6939; Abcam; Cambridge, UK) were added in turn for fluorescence staining. The distribution of red fluorescence in HCC tissues was observed under a 200-fold fluorescence microscope (Leica; Heidelberg, Germany), and the average fluorescence intensity of the target protein was calculated by Image-J image analysis system.

### Cell viability assay

After routine digestion, the cell density was adjusted to about 5 × 10^4^ cells/mL, and 100 μL cell suspension per well was inoculated in 96-well plate for 24 h. The control group and the reagent group were incubated for 0, 12, 24 or 48 h, respectively. Ang II and candesartan with different concentration gradients were added to the reagent group, while the control group was given the same volume of culture medium. After culture, 10 μL CCK-8 solution (#CK04; Dojindo; Kumamoto, Japan) was added into each well and mixed evenly. The absorbance value was read at 450 nm by microplate reader (Tecan; Mannedorf, Switzerland), and the sensitive dosage of HCC cells was screened out.

### Transwell experiment

HCC cells were placed in the Transwell upper chamber (Corning-Costar; Corning, NY, USA) to detect the migration ability of the cells. After adding serum culture medium to the lower chamber, they were set as control group, Ang II group and candesartan group, respectively. After 24 h of incubation, 0.1% crystal violet staining was used, and the average number of transmembrane cells was calculated under a 200-fold optical microscope in five non-repeated visual fields. In Transwell invasion test, Matrigel glue (#356234; BD Biosciences; Bedford, MA, USA) was used to coat the basement membrane of the chamber, and other operation steps were the same as those of migration test.

### Flow cytometry

Ang II and candesartan were used to stimulate hepatoma cells inoculated in six-well plates for 48 h, while NS administration was set as a control. The cells were collected with trypsin after the termination of the drug action by phosphate buffer washing, and then the rabbit anti-human AT1R primary antibody (Abcam; Cambridge, UK) and FITC-labeled fluorescent secondary antibody (#ZF-0311; ZSGB-BIO; Beijing, China) were added in turn. The percentage of AT1R positive HCC cells was detected and analyzed by FC500 flow cytometry (Beckman Coulter; Brea, CA, USA) and FlowJo software.

### Western blot analysis

The total proteins of HCC cells in each group were extracted and diluted to the appropriate concentration, then 10% SDS–polyacrylamide gel was prepared and placed into the electrophoresis tank. About 20 μL samples were added to each well respectively, and the electrophoresis was started with 70 V and 110 V voltage in turn. After electrophoresis, the protein in the gel was transferred to a PVDF membrane (#IPVH00010; Millipore; Bedford, MA, USA) in an electrotransfer tank, the membrane was blocked with bovine serum albumin, then anti-PLC-β1 and anti-CaM monoclonal primary antibodies and HRP-labeled secondary antibody were sequentially added, ECL reagent (#32209; Pierce; Rockford, IL, USA) was used for development and an ImageQuant LAS 4000mini chemiluminescence imager (GE Healthcare; Pittsburgh, PA, USA) was used for scanning. Finally, the effective value of protein expression was calculated by an Image-J image analysis system.

### RT-PCR and Real-time PCR

TRIzol reagent (#15596026; Invitrogen; Carlsbad, CA, USA) was used to extract total RNA from different groups of HCC cells, and the mRNA of AT1R, PLC-β1 or CaM was used as a template to synthesize complementary DNAs (cDNAs) using reverse transcription kit (#RR047A; Takara; Kyoto, Japan). After adding primers, they were placed in T100 PCR amplifier (Bio-Rad; Hercules, CA, USA) for cyclic amplification. The formed RT-PCR product was electrophoresed in a 2% agarose gel, stained with ethidium bromide (#E8751; Sigma-Aldrich; St. Louis, Mo, USA), and then photographed by a Bio-Rad gel imaging system. For Real-time PCR, 2^−ΔΔCT^ method was selected for real-time fluorescence quantitative detection, and then a standard curve was drawn and the relative expression of the target gene was analyzed [24].

### Statistical processing

SPSS 23.0 statistical software package (IBM SPSS; Chicago, IL, USA) was selected, and the data were analyzed by Student's t-test and one-way ANOVA. The results were expressed as mean ± standard deviation (SD), and the difference was statistically significant at P < 0.05.

## Results

### Expression levels of target proteins in serum and liver tissue of HCC patients

**Fig. 3:**
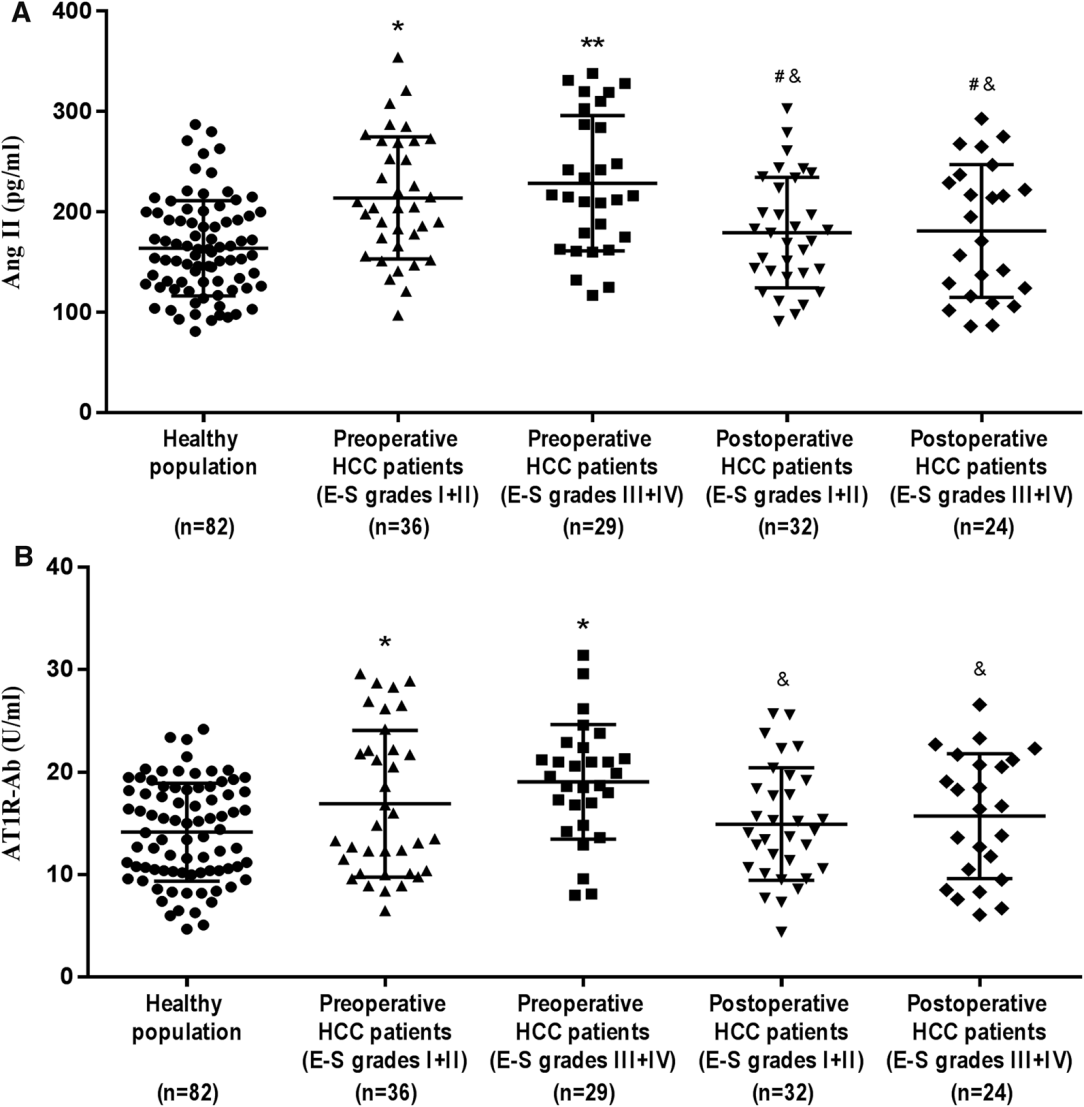

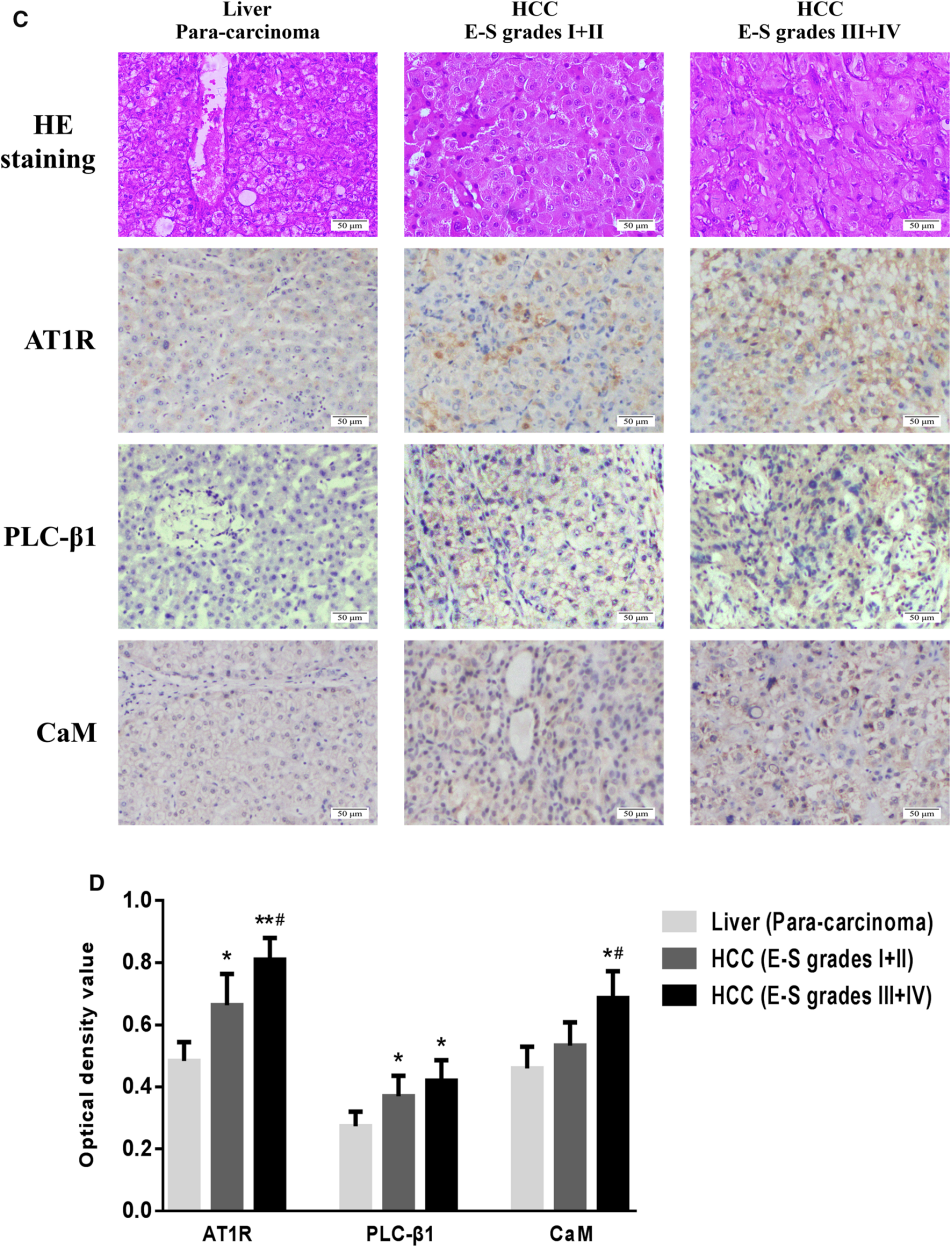
The expression levels of target proteins in serum and liver tissue of HCC patients. **A**, **B** ELISA was used to detect the levels of Ang II and AT1R-Ab in serum of HCC patients and normal people. There were 65 patients with HCC, 56 patients returned to the clinic 6 months after operation, and 82 normal people. *P  <  0.05, **P  <  0.01 VS normal population; ^#^P  <  0.05 VS preoperative HCC patients (E-S grades I + II); and P  <  0.05 VS preoperative HCC patients (E-S grades III + IV). **C**, **D** Representative IHC images and semi-quantitative analysis showed the expressions of AT1R, PLC-β1 and CaM in different liver tissues (magnification,  × 200). *P  <  0.05, **P < 0.01 VS liver para-carcinoma tissue; ^#^P  <  0.05 VS HCC tissue (E-S grades I + II)

The results of ELISA showed that the levels of Ang II and AT1R-Ab in serum of HCC patients before operation were significantly higher than those of normal people. The reexamination at 6 months after operation found that there were no statistical differences between the expressions of Ang II and AT1R-Ab in serum of patients and normal people, and the levels of Ang II in serum of patients after operation were obviously lower than those before operation (Fig. 3A, B). According to Edmondson-Steiner pathological grading method, the differentiation degree, nuclear/cytoplasmic ratio and atypia of cells in HCC specimens were evaluated, including 36 cases with grades I and II, and 29 cases with grades III and IV (Fig. 3C). Further detection by IHC showed that the expressions of AT1R, PLC-β1 and CaM in HCC tissues were significantly higher than those in para-carcinoma tissues, and with the increase of Edmondson-Steiner pathological grade, the levels of the above-mentioned target proteins in HCC specimens increased to different degrees (Fig. 3D).

### Correlation between AT1R, PLC-β1 and CaM and postoperative survival in patients with HCC

**Fig. 4:**
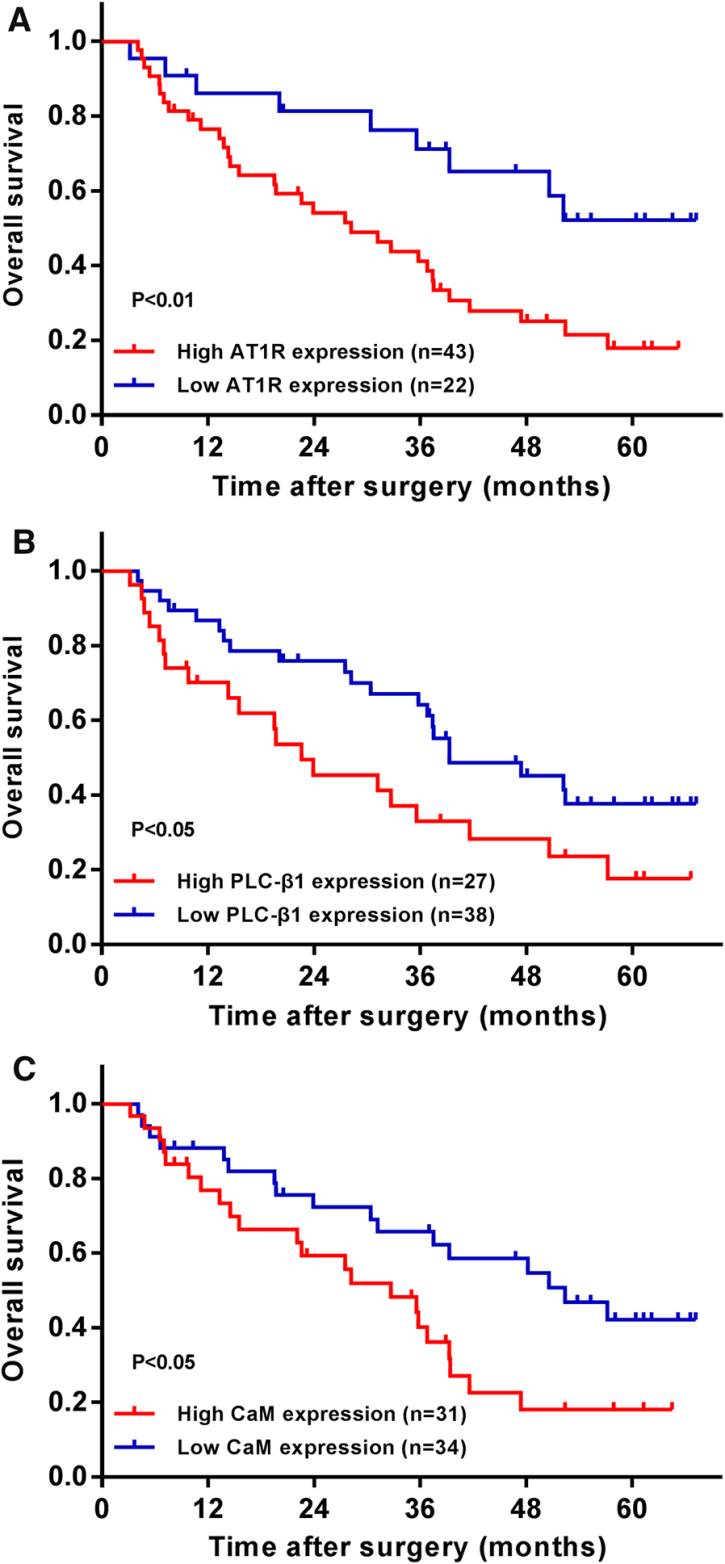
The influence of AT1R, PLC-β1 and CaM levels in liver tissue on the postoperative survival of HCC patients (n = 65). **A**–**C** Kaplan–Meier survival curve showed the correlation between target protein expression and postoperative survival time of HCC patients. P  <  0.05, P  <  0.01 VS high protein expression group

IHC was used to analyze the staining intensity of the target proteins and the proportion of positive cells in the HCC tissues of patients. The staining intensity score standard was 0 points for non-staining, 1 point for yellow, 2 points for pale brown and 3 points for yellowish-brown. The scoring criteria for the proportion of positive cells were as follows: 0 points for those patients with positive cells less than 5%, 1 point for those patients with 5–25% of positive cells, 2 points for those patients with 26–50% of positive cells, 3 points for those patients with 51–75% of positive cells and 4 points for those patients with positive cells more than 75%. The sum of the two scores was 0–3 for weak expression and 4–7 for strong expression. According to the above criteria, HCC patients were divided into high and low expression groups of AT1R, PLC-β1 and CaM for follow-up. Kaplan–Meier survival curve was drawn, and it was found that the postoperative survival time of patients with high expression of AT1R, PLC-β1 or CaM was significantly shortened, which are the risk factors affecting the prognosis of HCC (Fig. 4A–C).

### Effects of Ang II and candesartan on migration and invasion of HCC cells

**Fig. 5:**
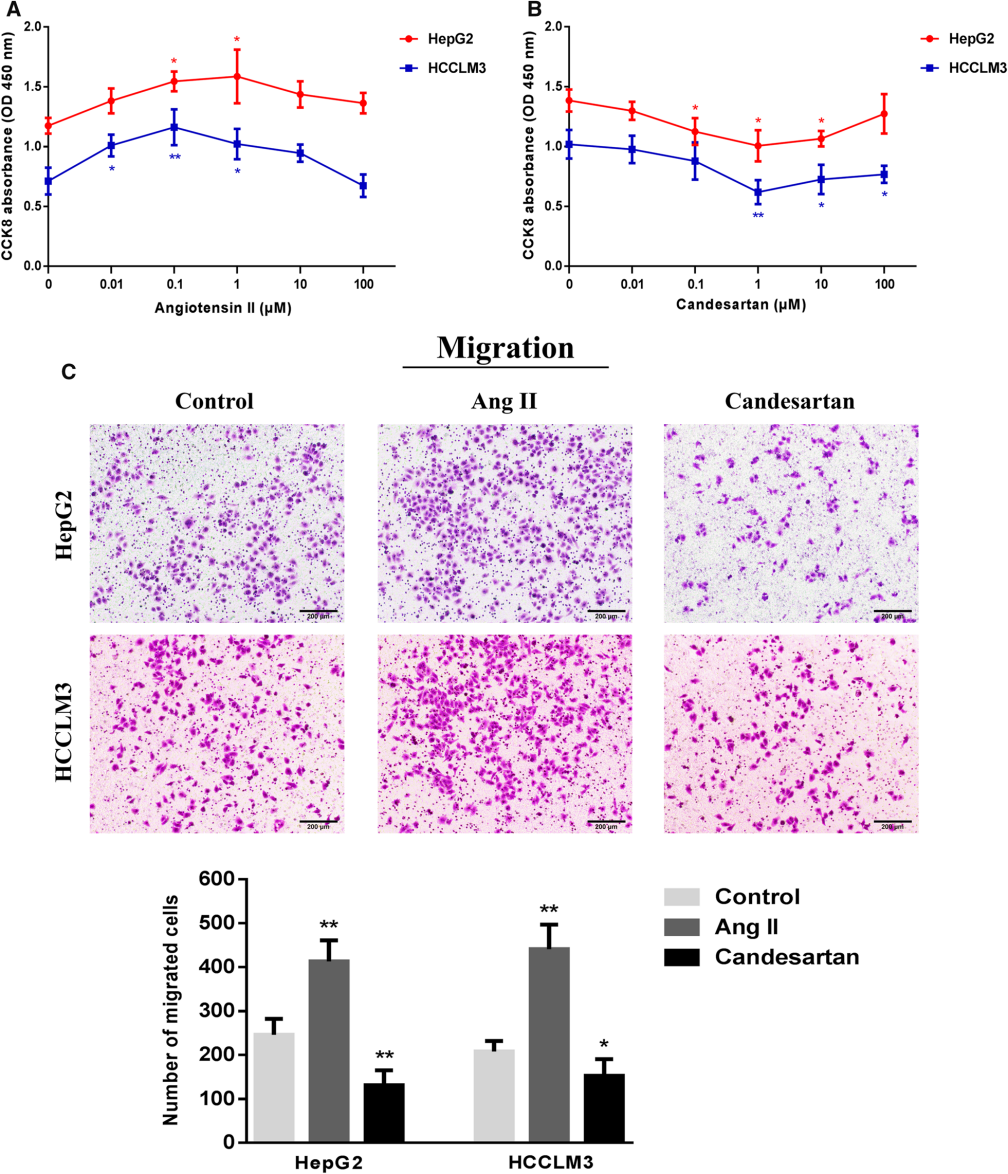

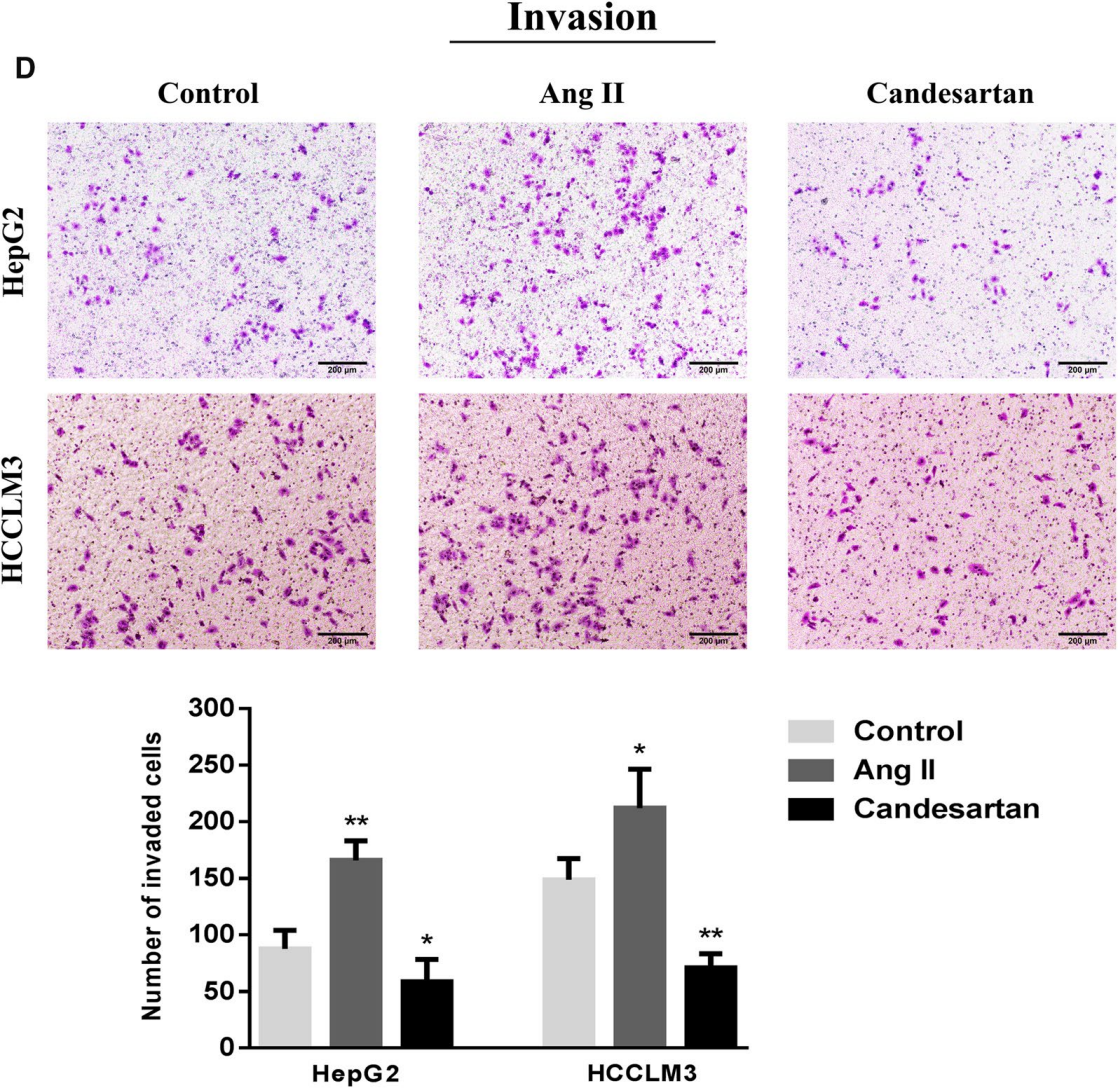
The role of Ang II and candesartan on the migration and invasion of HCC cells. **A**, **B** The CCK-8 absorbance values of HepG2 and HCCLM3 cells stimulated by Ang II and candesartan with different concentration gradients for 48 h. **C**, **D** Transwell assay was used to analyze the effects of Ang II and candesartan on the migration and invasion of HCC cells (magnification,  × 50). *P < 0.05, **P < 0.01 VS control group

Different concentration gradients (0.01, 0.1, 1, 10 and 100 μmol/L) of Ang II and candesartan were selected to stimulate HepG2 and HCCLM3 cells for 48 h, and the sensitive doses of Ang II and candesartan were screened by CCK-8 method. The results confirmed that 0.1 μmol/L Ang II and 1 μmol/L candesartan had the most significant effects on the growth of HCC cells (Fig. 5A, B). Transwell test was further used to detect the effects of Ang II and candesartan on the mobility of HCC cells. After 48 h treatment, it was found that Ang II could obviously promote the migration and invasion activity of HCC cells, while candesartan played a completely opposite role in this process (Fig. 5C, D).

### Changes in the expressions of AT1R, PLC-β1 and CaM during the migration and invasion of HCC cells

**Fig. 6:**
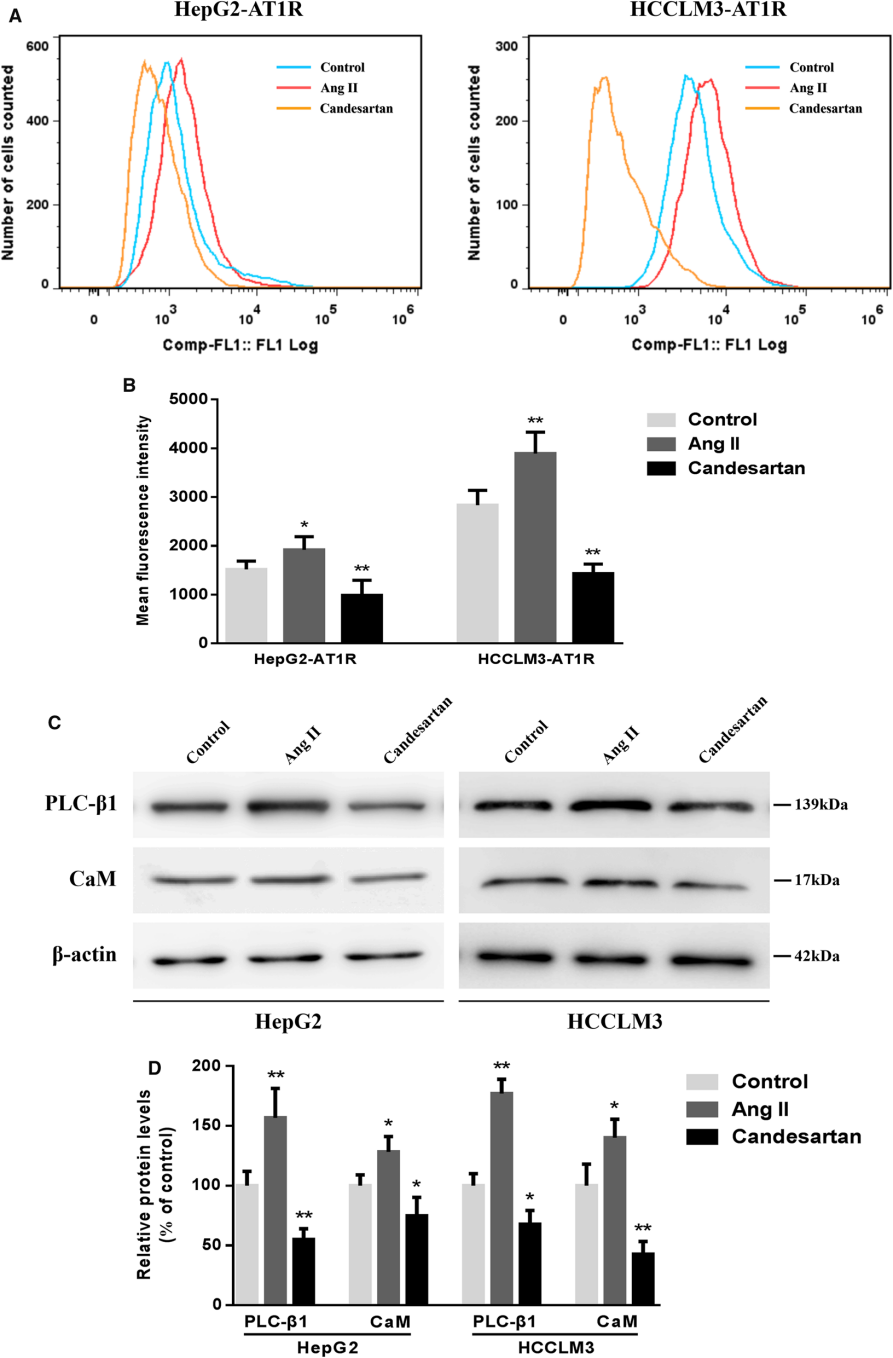
Effects of Ang II and candesartan on the expressions of AT1R, PLC-β1 and CaM in HCC cells. **A**, **B** FCM assay was used to detect the expression of AT1R on the surface of HepG2 and HCCLM3 cells stimulated by Ang II and candesartan for 48 h. **C**, **D** Representative western blot images and semi-quantitative analysis showed the differences in PLC-β1 and CaM levels in HCC cells between different drug groups. *P  <  0.05, **P  <  0.01 VS control group

The changes in the expression levels of the target proteins in the cells were detected when Ang II and candesartan affected the migration and invasion of HCC cells. Flow cytometry confirmed that the percentage of AT1R positive cells increased significantly after Ang II stimulated HepG2 and HCCLM3 cells for 48 h, while the percentage of AT1R positive cells in candesartan group decreased obviously (Fig. 6A, B). Furthermore, Western blot assay showed that the levels of PLC-β1 and CaM in HCC cells were closely related to the expression of AT1R on the cell surface, and the three signal proteins showed a consistent upward and downward trend under the action of Ang II and candesartan (Fig. 6C, D) (Additional file 1).

### Changes of target proteins and biological activity after transfection of PLC-β1 siRNA into HCC cells

**Fig. 7:**
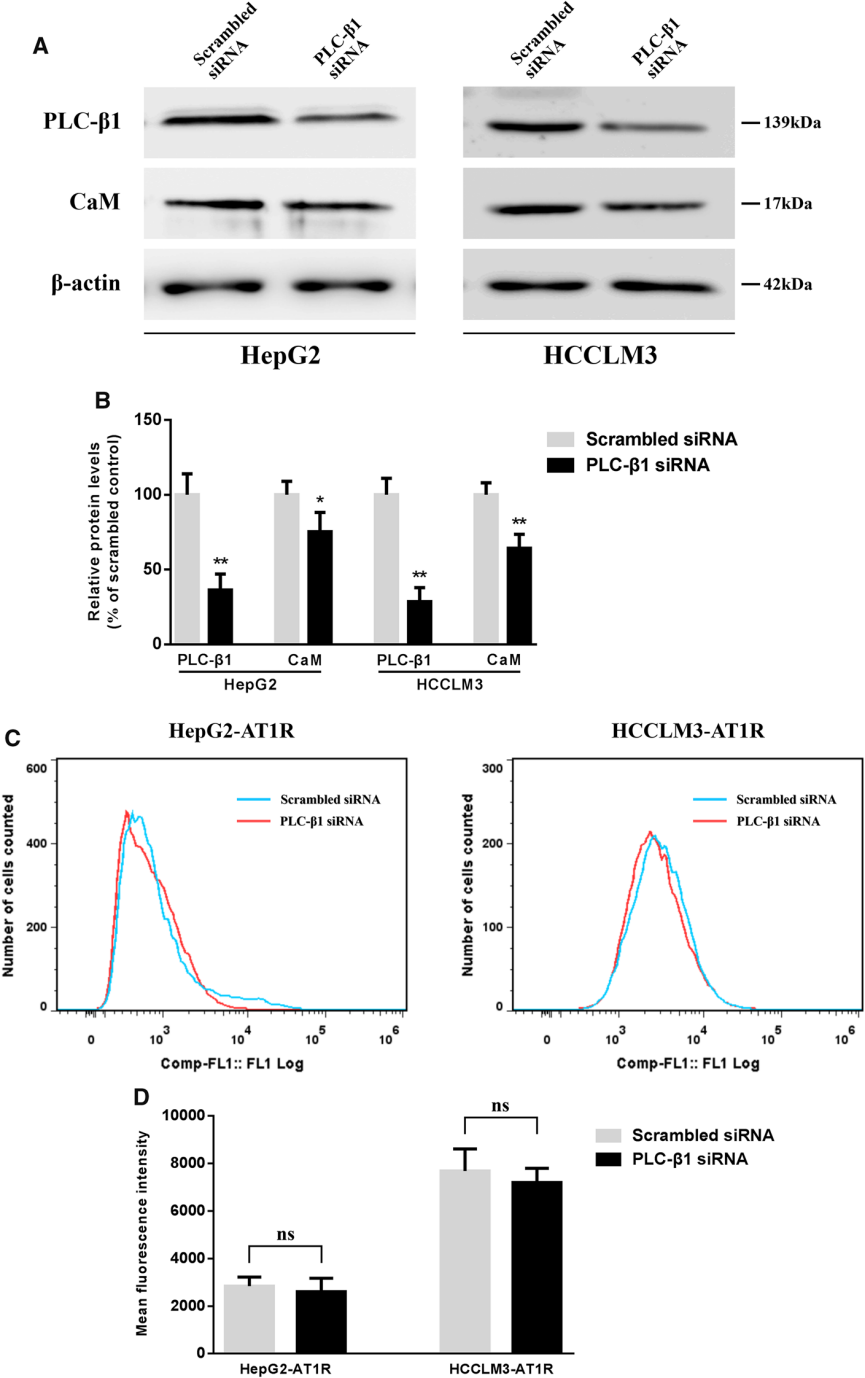

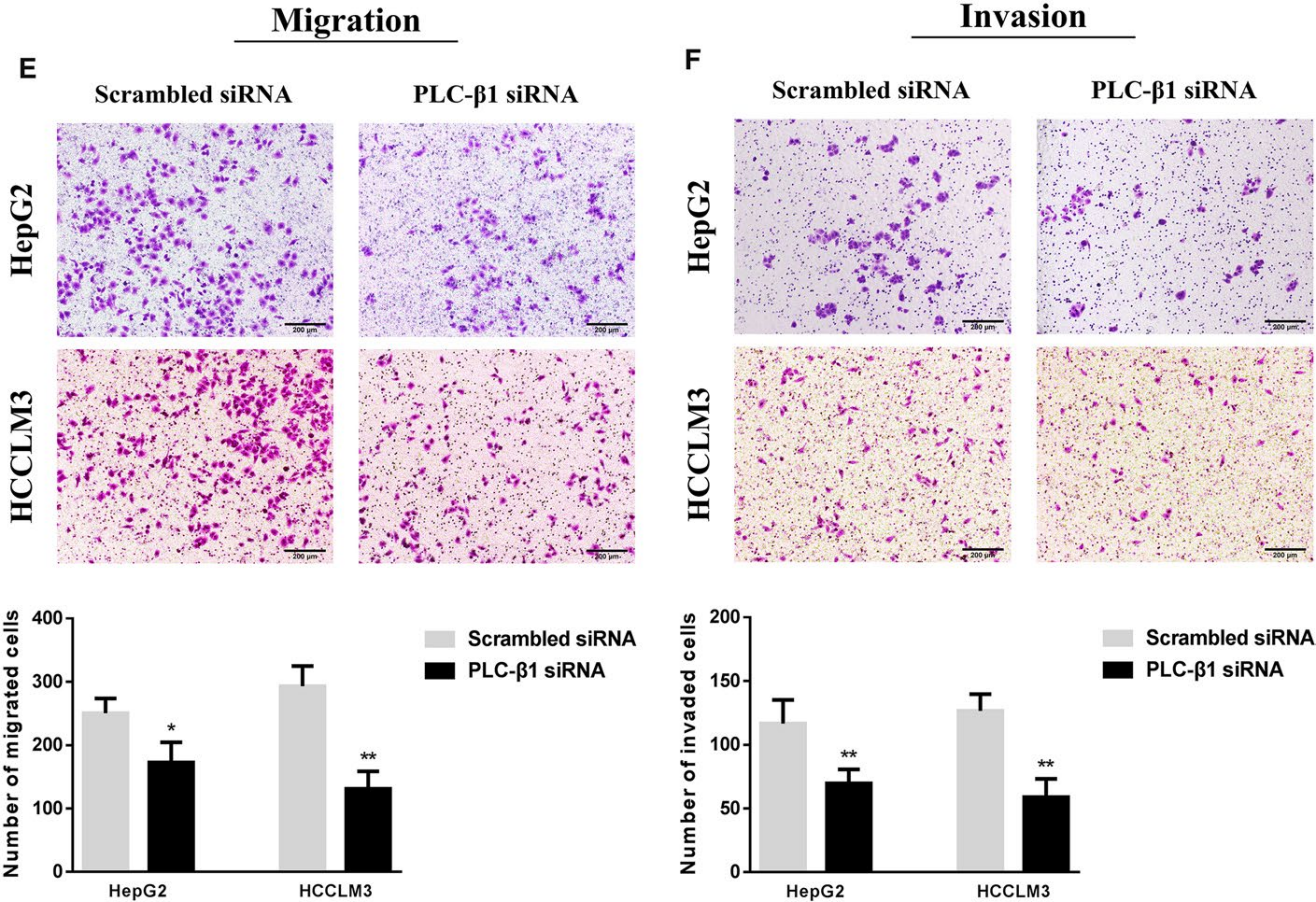
PLC-β1 siRNA interferes with the expression levels and biological activity of target proteins in HCC cells. **A**, **B** Western blot method was used to analyze the differences of PLC-β1 and CaM levels in HepG2 and HCCLM3 cells between different siRNA transfection groups. **C**, **D** FCM assay was used to detect the expression of AT1R on the surface of HCC cells transfected with different siRNA. **E**, **F** Representative Transwell images and quantitative analysis showed the changes in the migration and invasion activity of HCC cells transfected with different siRNA (magnification,  × 50). *P  <  0.05, **P  <  0.01 VS Scrambled siRNA group

PLC-β1 siRNA was used to interfere with HepG2 and HCCLM3 cells to establish HCC cell lines with low expression of PLC-β1. The results of western blot and flow cytometry showed that PLC-β1 and CaM levels in PLC-β1 siRNA group were significantly lower than those in scrambled siRNA group. There was no obvious difference in AT1R expression on the cell surface between the two groups, confirming that PLC-β1 siRNA transfection is effective and can regulate the expression of downstream CaM protein, but has no significant effect on the upstream AT1R level (Fig. 7A–D). Further Transwell experiment results showed that low expressions of PLC-β1 and CaM could obviously reduce the migration and invasion ability of HCC cells (Fig. 7E, F).

### Effects of Ang II and candesartan on the target proteins and biological activity in HCC cells transfected with PLC-β1 siRNA

**Fig. 8:**
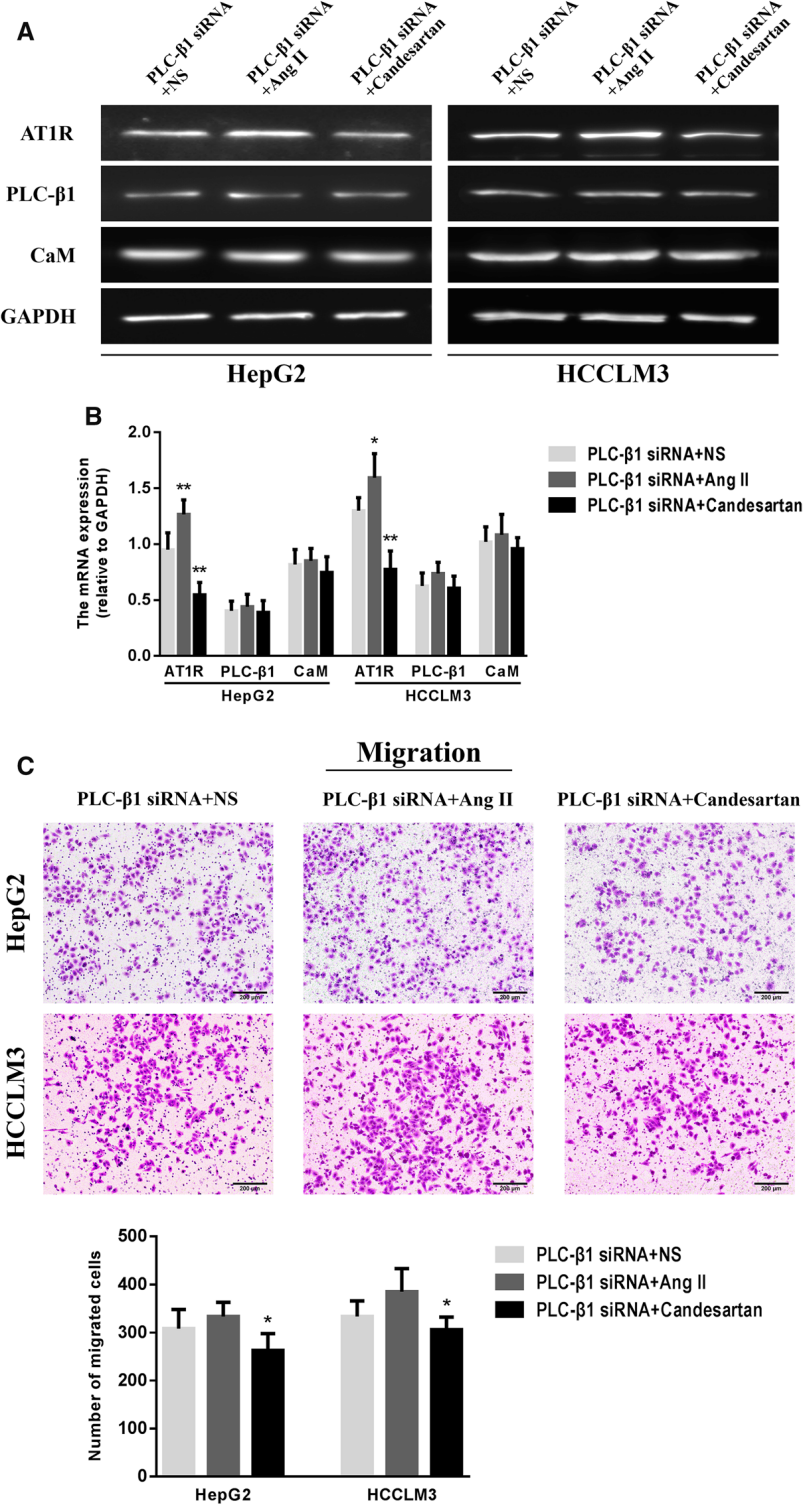

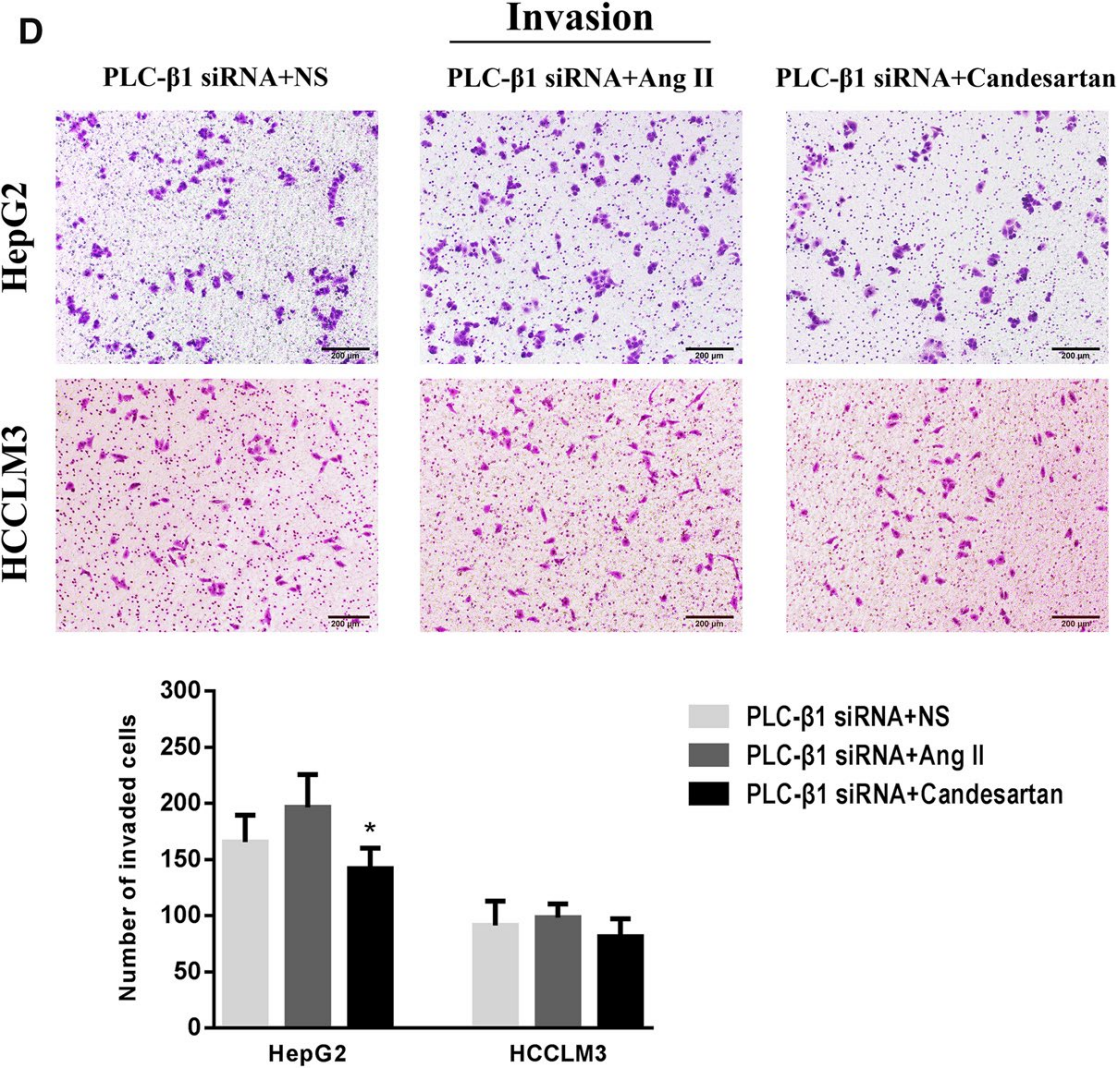
Effects of Ang II and candesartan on the expressions and biological activity of target proteins in HCC cells transfected with PLC-β1 siRNA. **A**, **B** RT-PCR and Real-time PCR were used to analyze the differences of AT1R, PLC-β1 and CaM levels in transfected HCC cells among different drug groups. *P < 0.05, **P < 0.01 VS PLC-β1 siRNA + NS group. **C**, **D** Representative Transwell images and quantitative analysis showed the changes in the migration and invasion activity of transfected HCC cells after medication (magnification,  × 50). *P < 0.05 VS PLC-β1 siRNA + Ang II group

Ang II and candesartan were used to stimulate HepG2 and HCCLM3 cells transfected with PLC-β1 siRNA for 48 h. RT-PCR and Real-time PCR detection showed that Ang II and candesartan had significant promotion and inhibition effects on AT1R mRNA expression in HCC cells, but had no obvious effect on PLC-β1 mRNA and CaM mRNA levels (Fig. 8A, B). The influences of Ang II and candesartan on the motility of HepG2 and HCCLM3 cells transfected with PLC-β1 siRNA were further analyzed by Transwell assay, and the results confirmed that the regulation of Ang II and candesartan on migration and invasion activity in HCC cells with low expressions of PLC-β1 and CaM was significantly weakened (Fig. 8C, D).

### Roles of Ang II and candesartan in the development of HCC in mice

**Fig. 9:**
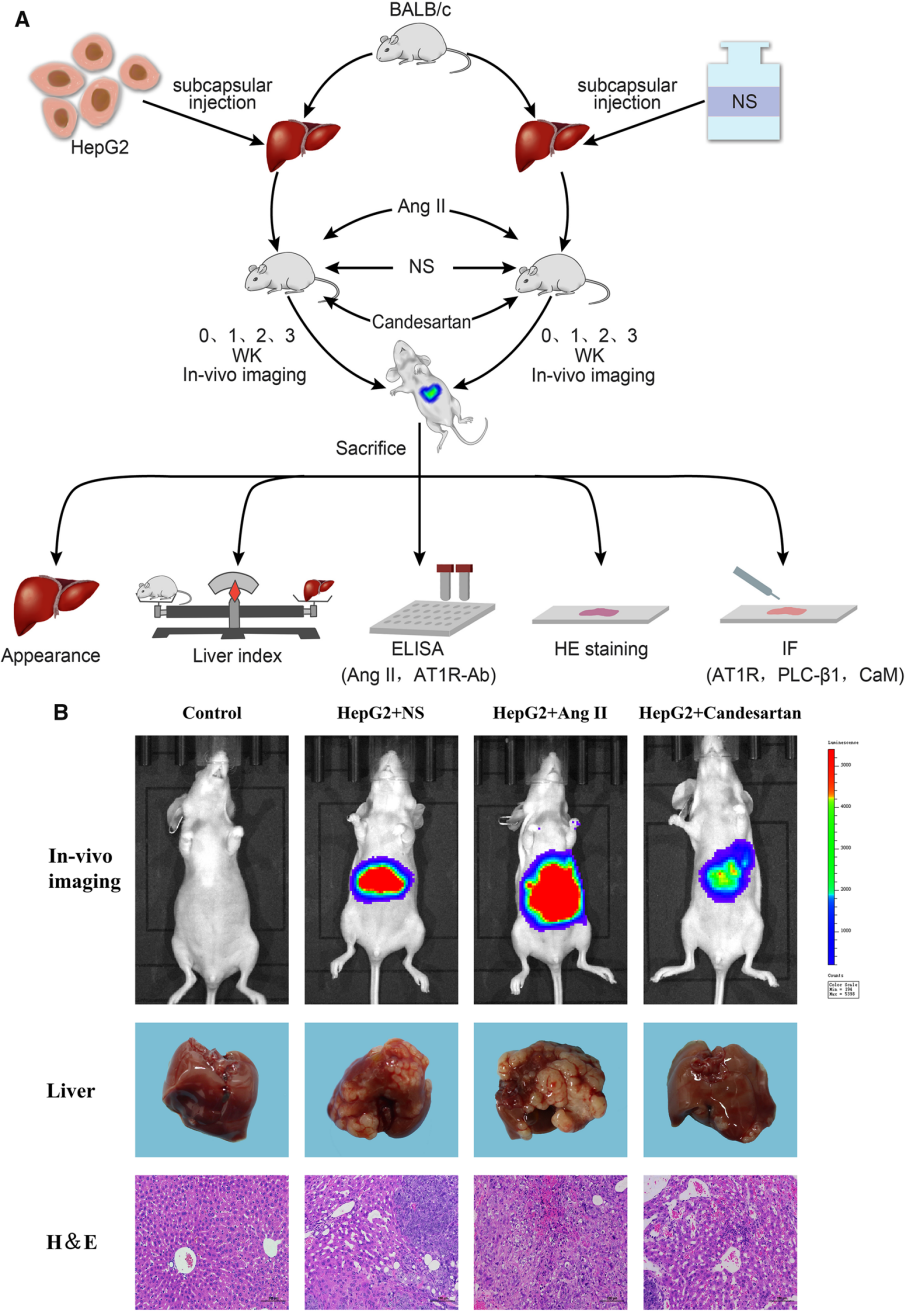

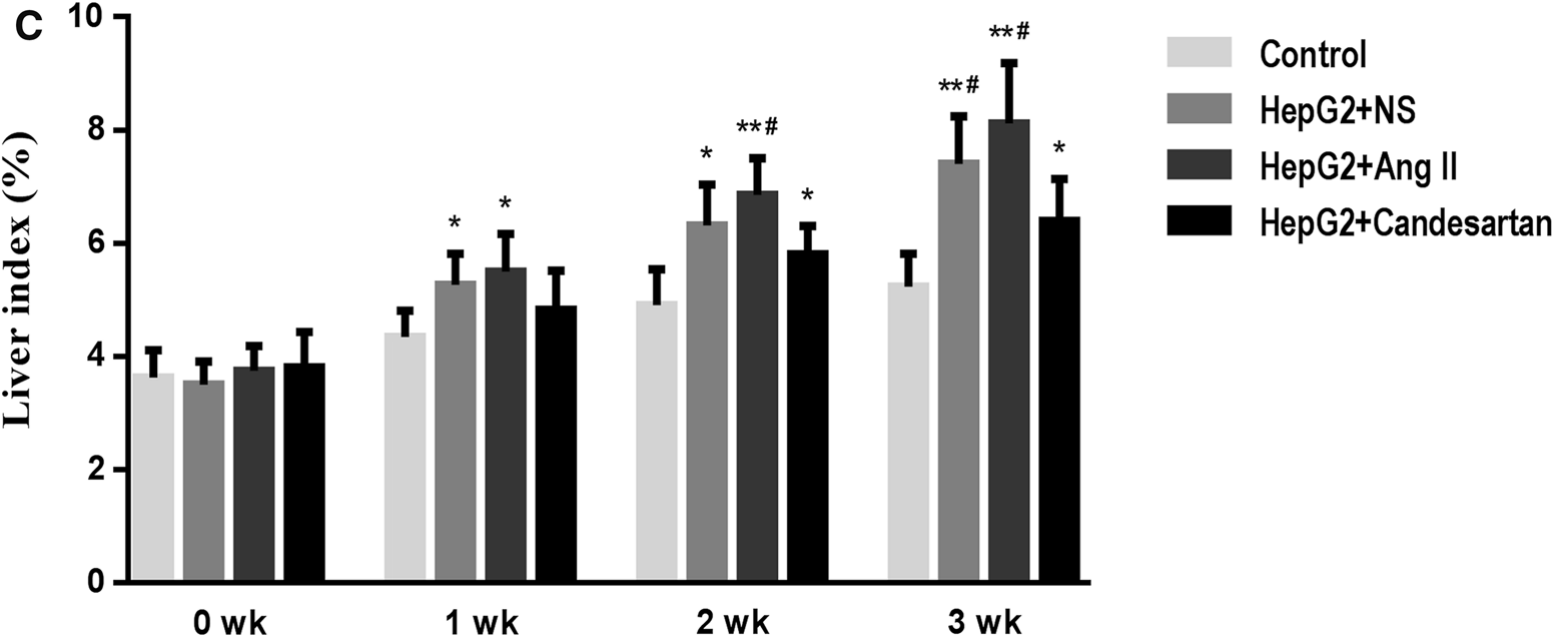
The role of Ang II and candesartan on the occurrence and development of mouse HCC. **A** Schematic diagram of the animal experimental study. **B** Differences in intraperitoneal tumor size, liver appearance and pathological structure after different drug interventions during the preparation of mouse HCC in situ model (HE magnification,  × 100). **C** The changes of liver index of mice in different medication groups at different stages of HCC model. Liver index = liver weight (g)/mouse body weight (g) × 100%. *P < 0.05, **P < 0.01 VS control group; ^#^P < 0.05 VS HepG2 + candesartan group

HepG2 cells were used to prepare mouse HCC in situ model, and then these mice were set up into Ang II group, candesartan group and NS group. In vivo imaging was performed 1 week, 2 weeks and 3 weeks after administration, respectively (Fig. 9A). It was found that Ang II could significantly increase the area and intensity of red fluorescence in mouse abdominal cavity, while candesartan played a completely opposite role. After the mice were sacrificed, the appearance and pathological characteristics of the liver were observed, and the differences in liver size, texture, tumor nodules, hepatic lobule structure and liver index of mice in each group were evaluated and calculated (Fig. 9B, C). It was confirmed that Ang II and candesartan had promotion and inhibition effects on the occurrence and development of mouse HCC respectively.

### Expression levels of target proteins in serum and liver tissue of HCC mice

**Fig. 10:**
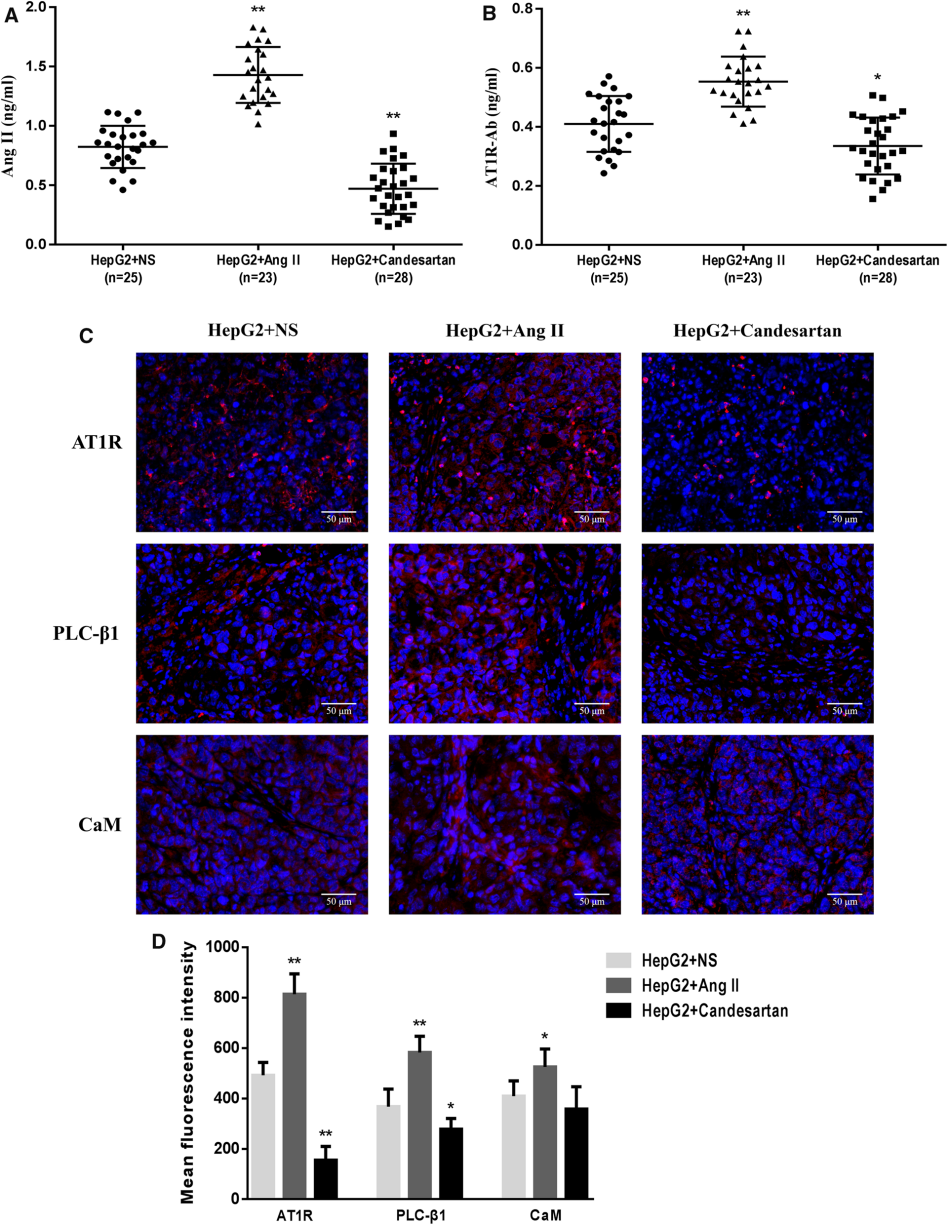
The expression levels of target proteins in serum and liver tissue of HCC mice (n = 25 in HepG2 + NS group, n = 23 in HepG2 + Ang II group, and n = 28 in HepG2 + candesartan group). **A**, **B** ELISA method was used to detect the levels of Ang II and AT1R-Ab in the serum of mice after different drug interventions during the preparation of HCC in situ model. **C**, **D** Representative immunofluorescence images and semi-quantitative analysis showed the expressions of AT1R, PLC-β1 and CaM in the liver tissue of mice in different medication groups (magnification,  × 200). *P < 0.05, **P < 0.01 VS HepG2 + NS group

ELISA assay was used to detect the expression levels of Ang II and AT1R-Ab in the serum of HCC mice in different drug groups. The results showed that compared with NS control group, Ang II could significantly increase the levels of Ang II and AT1R-Ab in the serum of mice, while the expressions of target proteins in the serum of mice in candesartan group was obviously decreased (Fig. 10A, B). Immunofluorescence method was used to analyze the differences in the expression levels of AT1R, PLC-β1 and CaM in liver specimens of HCC mice. It was found that the levels of AT1R, PLC-β1 and CaM in the liver tissue of Ang II group were increased to different degrees, while candesartan had a completely opposite effect on the expressions of the target proteins (Fig. 10C, D).

## Discussion

HCC is a common malignant tumor with high morbidity and mortality. Its occurrence and development involve a variety of pathophysiological processes. The process of tumor growth and metastasis can be regulated by interfering with the signaling pathway of HCC cells [25–28]. Many bioactive molecules in RAS system are closely related to the proliferation and invasion of HCC cells, so exploring the mechanism is of great significance for understanding the pathogenesis, prevention and treatment of HCC [29, 30]. This study found that the expressions of Ang II and AT1R-Ab in the serum of patients with HCC were significantly increased, and the levels of both in the serum decreased to different degrees after surgical treatment, suggesting that Ang II and AT1R-Ab may be the primary screening and prognostic indicators for HCC. Further immunohistochemical results showed that the levels of AT1R, PLC-β1 and CaM in HCC tissues were obviously higher than those in para-carcinoma tissues, and this trend became more prominent with the increase of Edmondson-Steiner pathological grade. The postoperative survival time of HCC patients with high expression of the above target proteins was significantly shortened. Therefore, AT1R, PLC-β1 and CaM may be risk factors affecting the formation and prognosis of HCC.

AT1R, PLC-β1 and CaM are considered to regulate the growth, metabolism, invasion and apoptosis of tumor cells, but their roles and relationships in the signal transduction process in HCC cells remain unclear. Therefore, it is of great significance to study their intervention mechanism in tumor metastasis pathway for finding new targets for HCC drugs [31–33]. Through cytological experiments, we found that 0.1 μmol/L Ang II and 1 μmol/L candesartan have the most significant effects on the motility of HCC cells, and regulating the expression level of AT1R on the cell surface is the main way to exert their biological effects. In this process, the change of AT1R level has an obvious impact on the expressions of downstream PLC-β1 and CaM proteins, and PLC-β1 siRNA was used to transfect HCC cells to further confirm the role of the above signaling molecules. The results demonstrated that the intervention ability of Ang II and candesartan in tumor cells with low expression of PLC-β1 and CaM was significantly weakened. Therefore, it is speculated that AT1R-mediated PLC-β1/CaM signaling pathway is an important way to upregulate the migration and invasion activity of HCC cells.

AT1R belongs to the GPCRs that has an important influence on the growth and metastasis of tumor cells, and exploring the correlation between AT1R and downstream PLC-β1/CaM signals in animal liver microenvironment is helpful to deepen the understanding of the mechanism of HCC occurrence and development [34–36]. In this study, HepG2 cells were successfully inoculated under the liver capsule of mice to induce the formation of HCC in situ. Ang II, candesartan and NS were administrated to observe the changes in the appearance and pathological structure of the liver. It was confirmed that Ang II can significantly promote the formation and progress of HCC in mice, and the candesartan showed completely opposite effects. By detecting the expression levels of the target proteins in the serum and liver tissue of mice in each group, we found that the levels of Ang II and AT1R-Ab in the serum of mice with higher progression of HCC were obviously increased, and the expressions of AT1R, PLC-β1 and CaM in the liver tissue of mice were also increased to different degrees. Therefore, the above signal proteins are all involved in the modeling process of mouse HCC and there is a certain correlation between them. This result further suggests that intervention of AT1R, PLC-β1 and CaM molecules may become a new research direction in the prevention and treatment of HCC in the future.

Common causative factors of HCC include hepatitis B, alcohol, aflatoxin and diabetes mellitus, but the specific pathogenesis is still open to debate. It is now believed that liver fibrosis, HCC cells proliferation, metastasis and tumor angiogenesis are important mechanisms that promote the formation and development of HCC [37]. Both hepatic stellate cells (HSCs) activation and extracellular matrix (ECM) deposition can induce the occurrence of liver fibrosis, while transforming growth factor-β (TGF-β)-mediated Smad and MAPK signals play an important role in this process [38–40]. TNF-α, Akt, mTOR, PI3K, ERK1/2 and IGF-1 are important signaling molecules affecting the growth of HCC, and activation of Raf protein by Ang II binding to AT1R was found to promote the proliferation of HCC cells [41]. However, losartan and other ARBs can inhibit the growth of HCC cells through inducing apoptosis [42]. The mechanisms associated with HCC metastasis are complex. Among them, angiogenesis is crucial for the development and invasion of tumor cells. Currently, NF-κB/MMP-9 and ROS/VEGF signals have been confirmed to play a significant role in the metastatic process of HCC [43, 44]. In contrast, various microRNAs such as miR-345, miR-219 and miR-126 are now gaining attention as important suppressors of HCC [45–47], and ACE2/Ang-(1–7)/MasR axis may exert a protective effect on the liver by counteracting the over-activated ACE/Ang II/AT1R signaling [48]. The results of this study suggest that the AT1R-mediated PLC-β1/CaM pathway can obviously promote the migration and invasion activity of HCC cells, and the effect of its regulation of pro-angiogenic factors such as VEGF, PDGF and EGFR on HCC angiogenesis will be the next research focus of our team.

## Conclusion

To sum up, AT1R, PLC-β1 and CaM are all important bioactive molecules that affect the formation and prognosis of HCC. Up-regulation of their expression levels can significantly promote the occurrence and development of mouse HCC, and mediating PLC-β1/CaM signaling pathway may be the main way of AT1R inducing migration and invasion of HCC cells. The results of this experiment lay a theoretical foundation for further exploring the mechanism of AT1R and its related signals regulating the pathophysiological functions of HCC in the future, and at the same time provide a basis for discovering new targets of HCC drugs and guiding patients to use drugs rationally in clinic, so it has important research value and application prospect.

## Supplementary Information

**Additional file 1.** The corresponding original images of Western blot.

## Data Availability

All data that support the findings of this study are available from the corresponding author upon reasonable request.

## Abbreviations

Ang II: Angiotensin II
PLC-β1: Phospholipase C-β1
CaM: Calmodulin
ARBs: Angiotensin receptor blockers
HSCs: Hepatic stellate cells
ECM: Extracellular matrix
TGF-β: Transforming growth factor-β
Smad: Small mother against decapentaplegic
MAPK: Mitogen-activated protein kinase
TNF-α: Tumor necrosis factor-α
mTOR: Mammalian target of rapamycin
PI3K: Phosphatidylinositol 3-kinase
ERK1/2: Extracellular signal-regulated kinase ½
IGF-1: Insulin-like growth factor-1
Raf: Rapidly accelerated fibrosarcoma
NF-κB: Nuclear factor-κappa B
MMP-9: Matrix metalloproteinase-9
ROS: Reactive oxygen species
VEGF: Vascular endothelial growth factor
ACE: Angiotensin converting enzyme
Ang-(1–7): Angiotensin-(1–7)
PDGF: Platelet derived growth factor
EGFR: Epithelial growth factor receptor
