# Supplementary material for: Effect of PLC-β1/CaM signaling pathway mediated by AT1R on the occurrence and development of hepatocellular carcinoma
Source: Cancer Cell Int. 2021 Nov 2;21:587. doi: 10.1186/s12935-021-02261-8 (PMC8561349; doi:10.1186/s12935-021-02261-8)

0riginal Western Blots

Fig 4 (HepG2/PLC-β1)


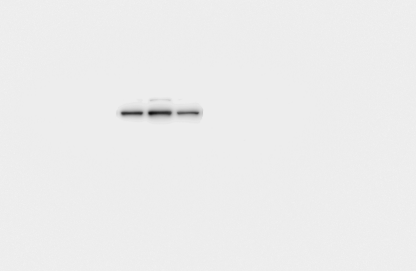


Fig 4 (HepG2/CaM)


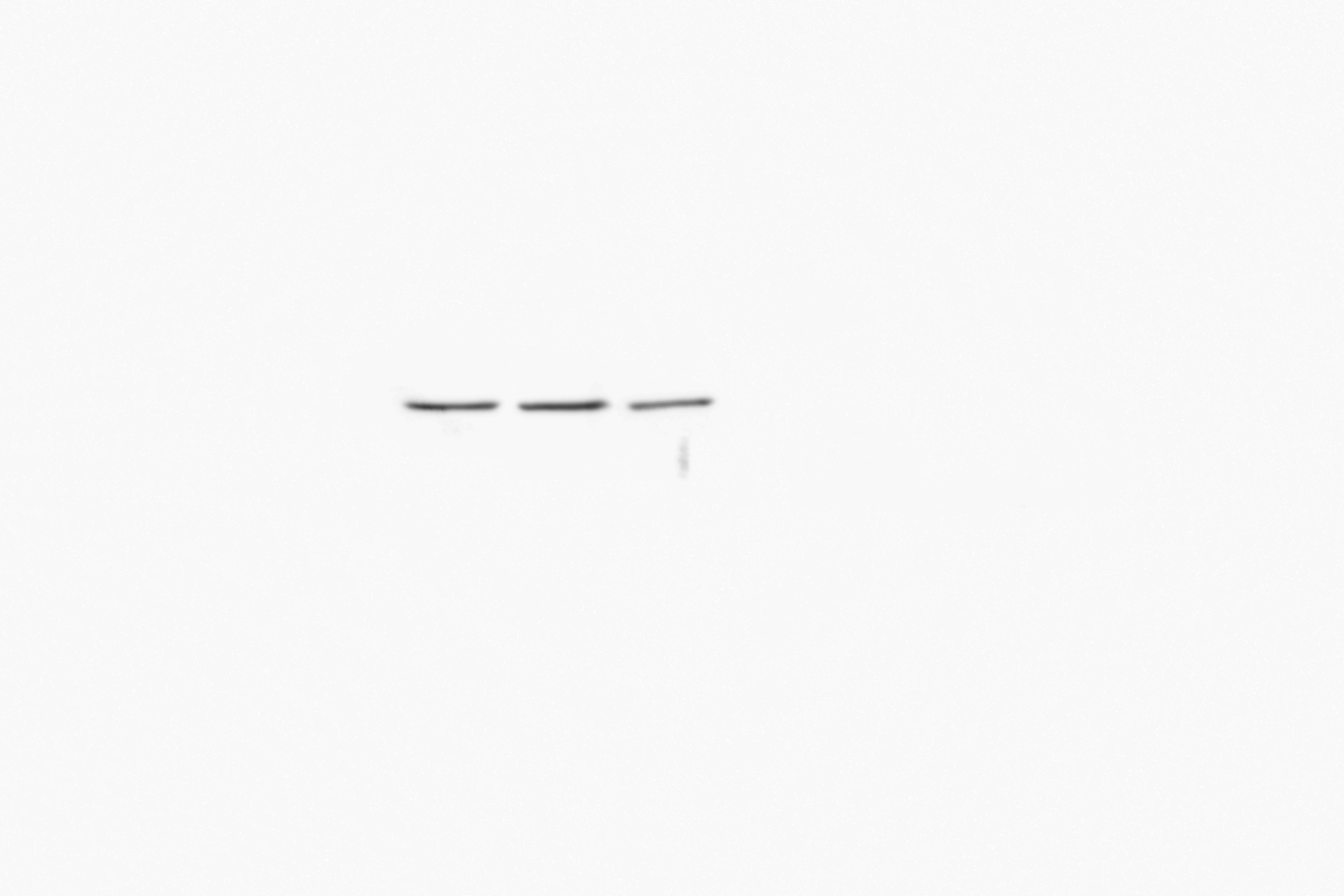


Fig 4 (HepG2/β-actin)


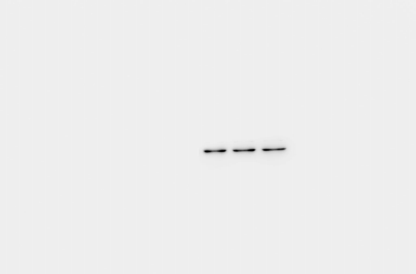


Fig 4 (HCCLM3/PLC-β1)


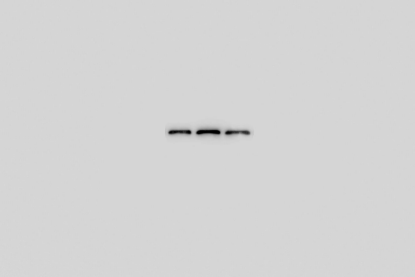


Fig 4 (HCCLM3/CaM)


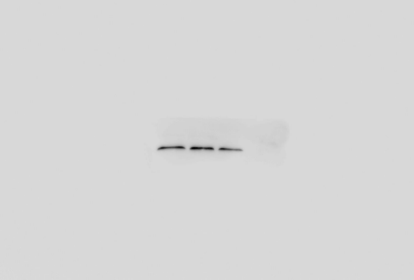


Fig 4 (HCCLM3/β-actin)


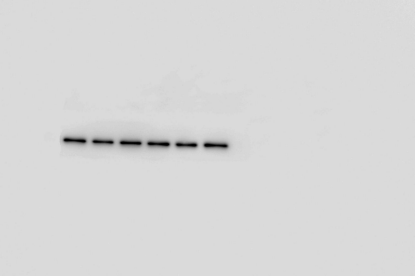


Fig 5 (HepG2+siRNA/PLC-β1)


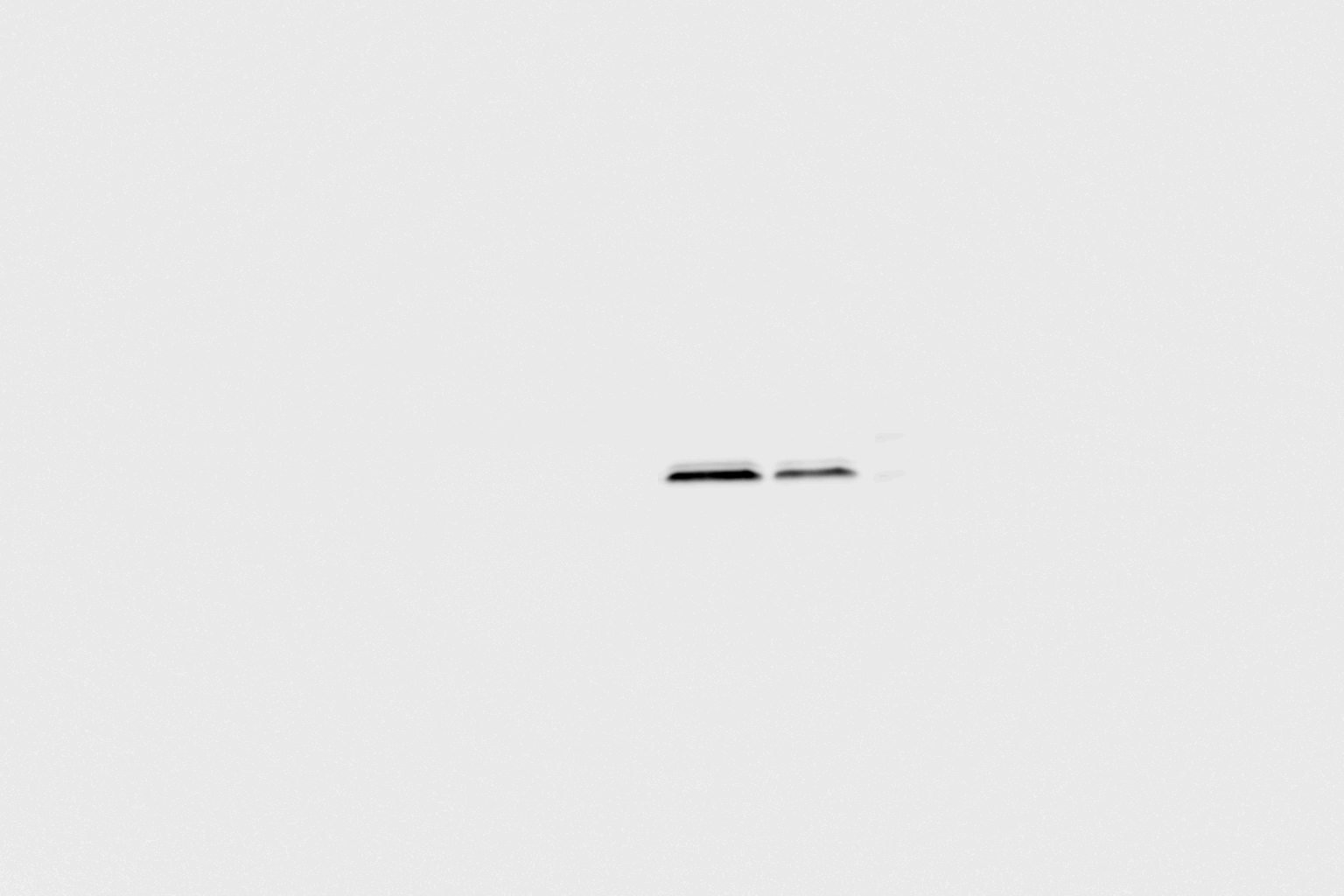


Fig 5 (HepG2+siRNA/CaM)


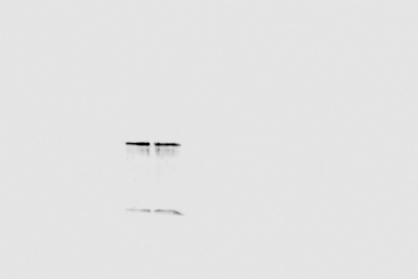


Fig 5 (HepG2+siRNA/β-actin)


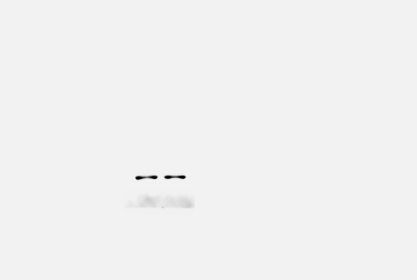


Fig 5 (HCCLM3+siRNA/PLC-β1)


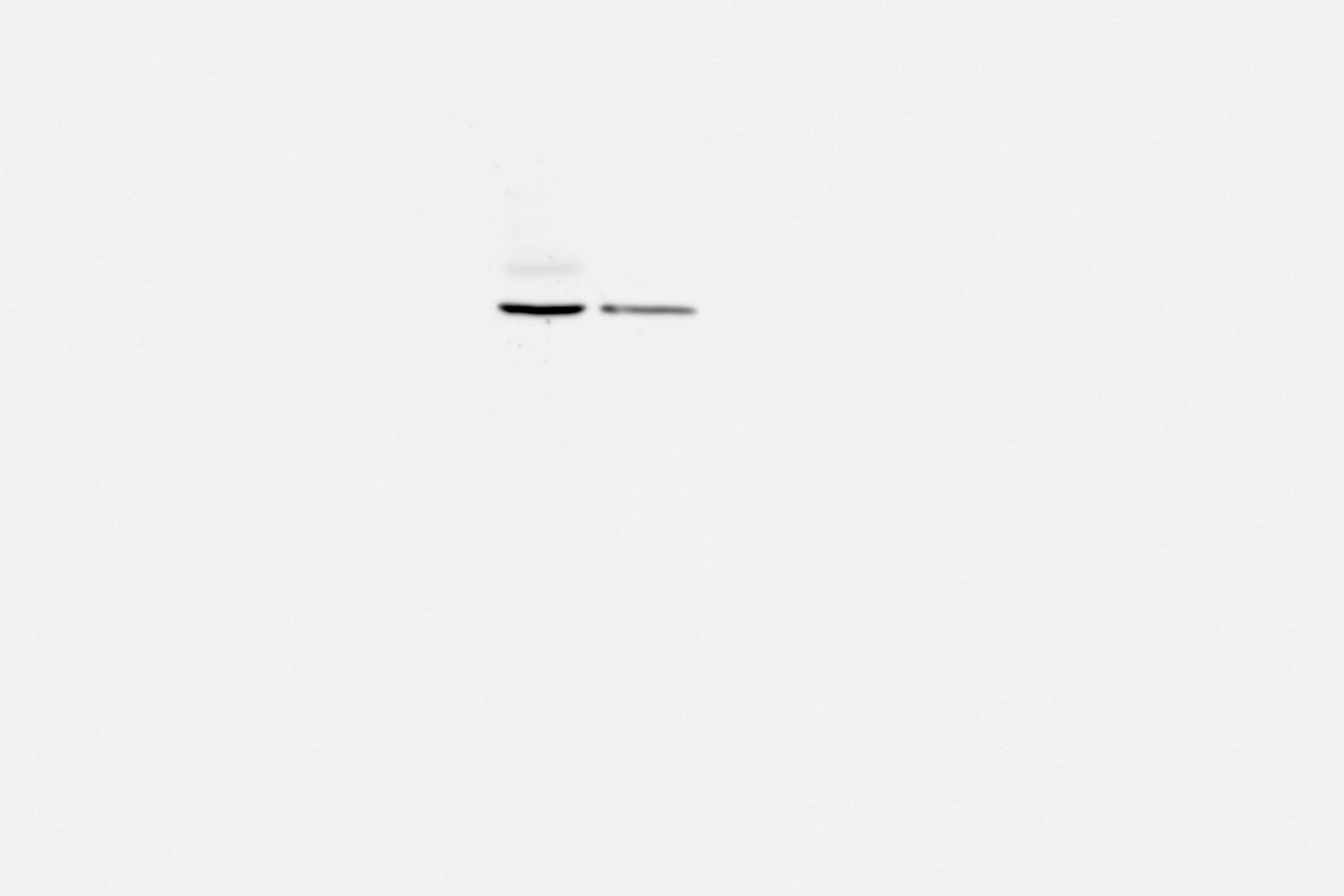


Fig 5 (HCCLM3+siRNA/CaM)


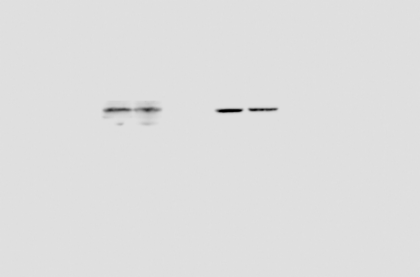


Fig 5 (HCCLM3+siRNA/β-actin)


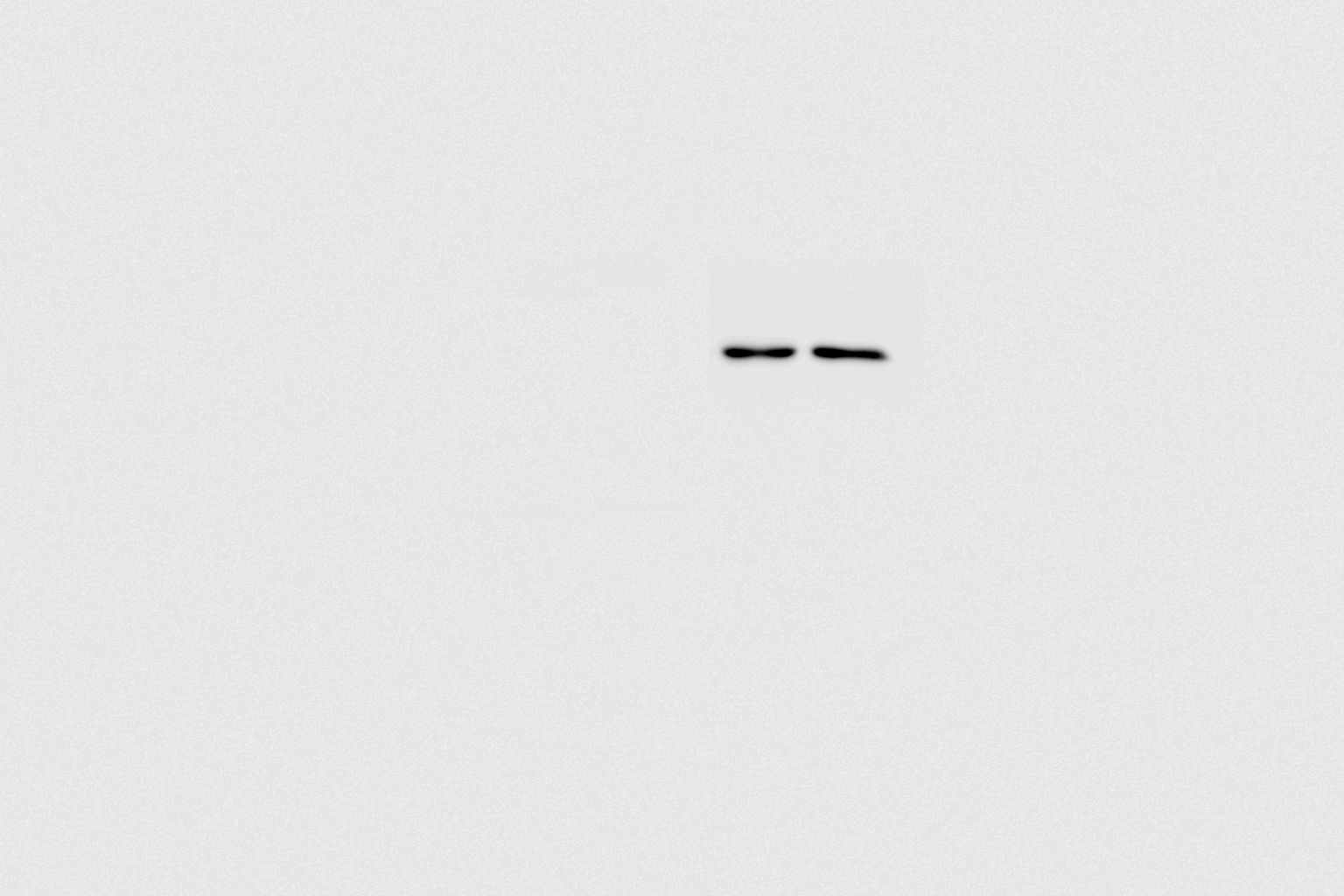

Supplement: Supplementary file 1 — Additional file 1. The corresponding original images of Western blot. [file 12935_2021_2261_MOESM1_ESM.doc]
